# Validity and reliability of the Dutch translation of the VISA-P questionnaire for patellar tendinopathy

**DOI:** 10.1186/1471-2474-10-102

**Published:** 2009-08-11

**Authors:** Johannes Zwerver, Tamara Kramer, Inge van den Akker-Scheek

**Affiliations:** 1 Center for Sports Medicine, University Center for Sport, Exercise and Health, University Medical Center Groningen, University of Groningen, PO BOX 30.001, 9700 RB Groningen, the Netherlands

## Abstract

**Background:**

The VISA-P questionnaire evaluates severity of symptoms, knee function and ability to play sports in athletes with patellar tendinopathy. This English-language self-administered brief patient outcome score was developed in Australia to monitor rehabilitation and to evaluate outcome of clinical studies. Aim of this study was to translate the questionnaire into Dutch and to study the reliability and validity of the Dutch version of the VISA-P.

**Methods:**

The questionnaire was translated into Dutch according to internationally recommended guidelines. Test-retest reliability was determined in 99 students with a time interval of 2.5 weeks. To determine discriminative validity of the Dutch VISA-P, 18 healthy students, 15 competitive volleyball players (at-risk population), 14 patients with patellar tendinopathy, 6 patients who had surgery for patellar tendinopathy, 17 patients with knee injuries other than patellar tendinopathy, and 9 patients with symptoms unrelated to their knees completed the Dutch VISA-P.

**Results:**

The Dutch VISA-P questionnaire showed satisfactory test-retest reliability (ICC = 0.74). The mean (± SD) VISA-P scores were 95 (± 9) for the healthy students, 89 (± 11) for the volleyball players, 58 (± 19) for patients with patellar tendinopathy, and 56 (± 21) for athletes who had surgery for patellar tendinopathy. Patients with other knee injuries or symptoms unrelated to the knee scored 62 (± 24) and 77 (± 24).

**Conclusion:**

The translated Dutch version of the VISA-P questionnaire is equivalent to its original version, has satisfactory test-retest reliability and is a valid score to evaluate symptoms, knee function and ability to play sports of Dutch athletes with patellar tendinopathy.

## Background

Jumper's knee (patellar tendinopathy) is an insertional tendinopathy most commonly affecting the patellar tendon's origin on the inferior pole of the patella [1]. Prolonged repetitive stress of the knee extensor apparatus can lead to this overuse tendinopathy of the patellar tendon in athletes from different sports [2]. Prevalence of patellar tendinopathy is especially high in jumping sports [3].

There is no consensus on what is the most appropriate treatment for patellar tendinopathy [4-6]. Exercise-based conservative treatment, including specific eccentric strengthening exercises, is considered to be useful in a rehabilitation program for patients with patellar tendinopathy [7]. However, further research is necessary to determine the most effective treatment strategies.

The VISA-P questionnaire has been introduced to quantify athletes' disability due to patellar tendinopathy, thereby facilitating research into this condition [8]. This self-administered brief questionnaire assesses symptoms, simple functional tests and ability to play sports. It has been proven to be a valid and reliable instrument for documentation of recovery from patellar tendinopathy [9,10]. It is claimed to determine clinical severity but is not a diagnostic tool.

The VISA-P questionnaire is an English-language questionnaire developed in Australia. It has already been translated into and adapted for a Swedish and an Italian version [11,12].

Aim of this study was to translate the questionnaire into Dutch and to study the reliability and validity of the Dutch version of the VISA-P.

## Methods

### The VISA-P questionnaire

The VISA-P questionnaire consists of eight questions [see Additional file 1]. Six out of eight questions rate pain during activities of daily living and simple functional tests on an inversed visual analogue scale from 0 to 10 points, with 10 representing optimal health. Two questions concern the ability to participate in sporting activities. The maximum VISA score for an asymptomatic athlete is 100 points.

### Translation procedure

The VISA Tendon Study Group in Australia was informed and gave their consent to a Dutch translation of the VISA-P questionnaire (Jill Cook, personal communication, 2008). The VISA group slightly modified the original version by changing the time periods in question 8 (Jill Cook, personal communication, 2008). It was therefore decided to translate the modified version.

The English VISA-P was translated according to the method described by Beaton et al. [13] This method recognises 5 stages: (1) translation, (2) synthesis, (3) back translation, (4) expert committee review and (5) pre-testing. Two students (one informed, one uninformed) independently translated the questionnaire into Dutch (stage 1). At stage 2, a synthesis was made of these two translations. Back translation (stage 3) was done independently by two native English speakers fluent in Dutch, one with a medical background and one without. The expert committee consisting of two translators from stages 1 and 3, a sports medicine physician and a human movement scientist/epidemiologist drafted the final version (stage 4), which was pre-tested on eight persons.

### Subjects

All subjects were asked for demographic characteristics (gender, age, height, weight and sport hours per week).

#### - Reliability

To assess test-retest reliability, 99 students filled out the Dutch VISA-P twice, at an interval of 2.5 weeks.

#### - Validity

To determine discriminative validity of the Dutch VISA-P, 89 persons completed it. They were divided into six groups: (1) 18 healthy students, (2) 15 competitive volleyball players (at-risk population), (3) 14 patients with the of diagnosis patellar tendinopathy, (4) 6 patients who had surgery for patellar tendinopathy 6 months before, (5) 17 patients who had knee injuries other than patellar tendinopathy, and (6) 19 patients with symptoms unrelated to their knees. All patients were treated at the Center for Sports Medicine, University Medical Center Groningen, The Netherlands. The study was conducted according to the regulations of the Medical Ethical Committee at University Medical Center Groningen.

### Statistics

Descriptive statistics (mean, SD) were used to describe the subject characteristics.

#### - Reliability

Internal consistency was determined with Cronbach's alpha. A principal component analysis with varimax rotation was carried out to analyse the factor structure (eigen value above 1). Intraclass correlation coefficients (ICCs) were calculated to analyse test-retest reliability, and Bland and Altman plots were constructed.

#### - Validity

Differences between the six groups were analysed with an ANOVA. The Bonferroni method was applied to correct for multiple testing in the post-hoc analyses. All statistical analyses were carried out using SPSS version 16.0. A significance level of 5% was applied.

## Results

### Translation

The expert committee agreed with the translation and back-translation. Only the translation of the words in questions 4 and 5 was debated. Dutch athletes often use the English words 'lunge' and 'squatting' for these specific exercises instead of the Dutch translation (*uitvalspas *and *hurkbeweging*). Therefore it was decided to include both the English word and the Dutch translation. Pre-testing did not reveal any difficulties. For the Dutch VISA-P see Additional file 2.

### Subjects

#### - Reliability

**Figure 1:**
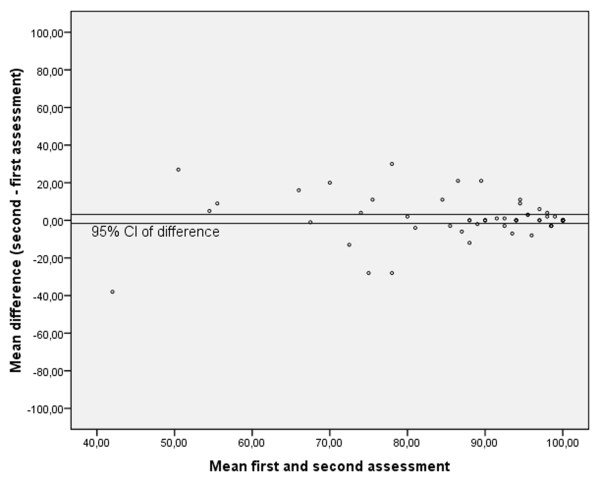
**Bland & Altman plot**.

Of the 99 students who completed the VISA-P the first time, 71 filled it in the second time. The mean VISA-P score of the 28 drop-outs did not differ significantly from that of the first assessment of the 71 students. Of these 71 students, 53 (75%) were female. Mean age was 19.2 (± 0.9) years, mean height 1.75 (± 0.07) m, mean weight 67.4 (± 10.0) kg, and mean hours of sports activities per week 5.36 (± 4.1). The mean VISA-P score (± SD) was 89.5 (± 14.3) and 90.3 (± 14.2) at the first and second assessments, respectively. The ICC between the first and second assessments was 0.74 (P < 0.001). When looking at the individual questions, five out of eight questions had an ICC > 0.60 (range 0.45–0.82). Bland and Altman plot (Figure 1) shows that zero lies within the 95% CI of the mean difference, indicating that no bias had occurred.

The Cronbach's alpha was 0.73 for the first and 0.71 for the second assessment. The principal component factor analysis yielded a two-factor structure, explaining 64.5% of the total variance. The question about sitting pain-free had the lowest factor score (0.53). Forcing a three-factor structure, explaining 74.6% of total variance, resulted in one component with five questions (pain during activities), a second component with two questions (physical activity participation), and a third component with only one question (pain during sitting). The lowest factor score was 0.68 (see Table 1).

**Table 1:** Principal component analysis with varimax rotation, forced three-factor structure.

	Component		
	
	1	2	3
Q1	0.21	0.05	**0.94**

Q2	**0.89**	0.11	0.15

Q3	**0.80**	-0.24	0.10

Q4	**0.75**	0.42	0.14

Q5	**0.69**	0.48	0.17

Q6	**0.71**	0.37	0.33

Q7	0.01	**0.78**	-0.09

Q8	0.24	**0.68**	0.35

#### - Validity

Mean VISA-P scores (± SD) and characteristics of the participants in the six different groups are displayed in Table 2. ANOVA revealed a significant difference between the six groups (F = 10.7, p < 0.001). When looking at the eight questions separately, on questions 1 and 3 no significant difference was seen (p = 0.20 and 0.16, respectively). Post-hoc analyses (Bonferroni correction) revealed that the mean VISA-P score of the group with patellar tendinopathy differed significantly from that of the healthy students group and the elite volleyball players (at-risk group), but not from the other three groups.

**Table 2:** VISA-P scores and demographic characteristics (means (SD)) of the participants of the validity study.

	**Healthy students**	**At-risk population**	**Injury other than knee**	**Knee injury**	**Patellar tendinopathy**	**Surgery for patellar tendinopathy**	**Total**
**N**	18	15	19	17	14	6	89

**VISA-P**	95.3 (8.8)	88.6 (11.1)	76.6 (24.3)	61.9 (24.1)	58.2 (18.9)	56.0 (20.9)	75.3 (23.6)

**Male (%)**	7 (38.9)	8 (53.3)	4 (21.1)	11 (64.7)	11 (78.6)	5 (83.3)	46 (51.7)

**Age (yrs)**	20.0 (1.5)	25.2 (4.7)	19.2 (1.2)	24.7 (4.5)	25.1 (3.7)	32.5 (2.9)	23.0 (4.7)

**Height (m)**	1.76 (0.08)	1.87 (0.08)	1.77 (0.07)	1.84 (0.09)	1.85 (0.09)	1.81 (0.08)	1.81 (0.09)

**Weight (kg)**	69.9 (9.5)	82.0 (12.6)	69.2 (12.3)	82.1 (12.1)	80.5 (12.4)	93.3 (1.5)	77.0 (13.1)

**Sport (hours/week)**	5.1 (3.3)	8.0 (2.9)	5.6 (4.4)	3.8 (3.1)	4.5 (3.1)	1.0 (1.7)	5.2 (3.7)

## Discussion

### Translation

We feel confident that the translated Dutch VISA-P questionnaire is linguistically equivalent to the original version, since our study shows that the VISA-scores of both healthy subjects (95) and athletes with patellar tendinopathy visiting a sports medicine clinic (58) are comparable with the results of Visentini (95 and 55 respectively) [8]. Also, the expert translation committee judged the original and translated versions to be congruent.

#### - Reliability

Over a time interval of 2.5 weeks, the Dutch version of the VISA-P score showed satisfactory test-retest reliability (ICC = 0.74). This is slightly lower than in previous studies [8,11,12], which had much shorter test-retest intervals, ranging from 1 hour to 1 week. We decided to take a longer time interval in order to prevent participants from copying the VISA-P from memory. A time interval of two weeks or more is commonly used in reliability studies. We are aware that a limitation of this study is that the test-retest reliability was investigated in asymptomatic students. One could argue that testing reliability in athletes with patellar tendinopathy would have been more appropriate. However, in the reliability study of Frohm the majority of participants (66%) who were asked to fill out the VISA-P questionnaire were asymptomatic too [11]. No differences with regard to reliability were described between symptomatic and asymptomatic participants, therefore we believe that the reliability of the Dutch VISA-P questionnaire found in this study also applies to athletes with patellar tendinopathy.

#### - Validity

The mean VISA-P score varied significantly between the groups of healthy and injured subjects. As mentioned before, the VISA-P scores in this study were comparable with those from the original study of Visentini [8]. Healthy volunteers had VISA-P scores of 95 in both studies, and athletes with patellar tendinopathy also had comparable scores of 55 and 58. The VISA-P score of volleyball players (population at risk) in this study was within the same range as the score of the basketball players in the original publication, 89 and 92 respectively. Patients with other knee injuries (62) and even subjects with other injuries not related to the knee (77) also had low scores compared to healthy subjects. This indicates that the VISA-P is indeed not suitable for diagnostic use.

The VISA-P was developed to monitor – by intra-individual comparisons – the effectiveness of treatment and rehabilitation programmes in athletes with patellar tendinopathy as well as for research purposes to facilitate group comparisons. Although this study was not designed to investigate the sensitivity of the VISA-P score for change, there seems to be sufficient range between healthy subjects and athletes with patellar tendinopathy to detect differences. On a maximum of 100 points, healthy subjects scored 95 points compared to 58 in athletes with patellar tendinopathy.

Translation of the VISA-P questionnaire into the Dutch language now makes it possible to compare results from international studies with the Dutch situation. For example, in this study subjects who underwent surgery because of their patellar tendinopathy scored 'only' 56 points. In our department athletes only proceed to surgery if conservative treatment has failed and they still have pain during daily activities, experience painful functional tests and are unable to participate in sports at the desired level (VISA-P score approximately < 40). This is in line with previous findings by Bahr, who found a mean VISA-P score of 58 in patients who had a patellar tenotomy for six months (and a pre-surgical score of 31) [14].

Our results show that the Dutch version of the VISA-P score conveys the same information as that gathered by other international versions of this questionnaire. This validated and reliable questionnaire will not only facilitate patellar tendinopathy research in the Netherlands, it also allows international comparison.

## Conclusion

The results of the present study indicate that the translated Dutch version of the VISA-P questionnaire is equivalent to its original version, has satisfactory test-retest reliability, and is a valid score to evaluate symptoms, functional tests and ability to play sports of Dutch athletes with patellar tendinopathy.

## List of abbreviations

VISA-P: Victorian Institute of Sports Assessment – Patellar questionnaire

## Competing interests

The authors declare that they have no competing interests.

## Authors' contributions

JZ conceived the study, participated in its design and coordination, and drafted the manuscript. TK collected the data, performed the statistical analysis and helped to draft the manuscript. IA participated in the design of the study and helped conduct the statistical analysis and draft the manuscript. All authors read and approved the final manuscript.

## Pre-publication history

The pre-publication history for this paper can be accessed here:



## Supplementary Material

Additional file 1 **The VISA-P questionnaire**. The VISA-P questionnaire in English. Click here for file

Additional file 2 **The translated Dutch VISA-P questionnaire**. The Dutch version of the VISA-P questionnaire after translation. Click here for file
