# Supplementary material for: Validity and reliability of the Dutch translation of the VISA-P questionnaire for patellar tendinopathy
Source: BMC Musculoskelet Disord. 2009 Aug 11;10:102. doi: 10.1186/1471-2474-10-102 (PMC2734752; doi:10.1186/1471-2474-10-102)
Supplement: Additional file 1 — The VISA-P questionnaire. The VISA-P questionnaire in English. [file 1471-2474-10-102-S1.pdf]

Name: ________________________________ Date: ___________

The Victorian Institute of Sport Assessment Score

1. For how many minutes can you sit pain free?

0 – 15 mins	15 – 30 mins	30 – 60 mins	60 – 90 mins	90 – 120 mins	> 120 mins
☐	☐	☐	☐	☐	☐
0	2	4	6	8	10

2. Do you have pain walking downstairs normally?

Severe Pain / Unable												No pain
	0	1	2	3	4	5	6	7	8	9	10	

3. Do you have pain at the knee with full active non-weight bearing knee extension?

Severe Pain / Unable												No pain
	0	1	2	3	4	5	6	7	8	9	10	

4. Do you have pain when doing a lunge?

Severe Pain / Unable												No pain
	0	1	2	3	4	5	6	7	8	9	10	

5. Do you have problems when squatting?

Severe Problems / Unable												No pain
	0	1	2	3	4	5	6	7	8	9	10	

6. Do you have pain during or immediately after doing 10 single leg hops?

Severe Pain / Unable												No pain
	0	1	2	3	4	5	6	7	8	9	10	

7. Are you currently undertaking all aspects of normal training or activity?

0	☐	No. Not at all.
4	☐	Modified training or activity.
7	☐	Full training / competition but not at same level as when symptoms began.
10	☐	Competing at the same level as symptoms began.

8. This question has 3 parts - please answer one part only

- If you have no pain while being active or playing sport → answer **Q8a only**.
- If you have pain while active or playing sport but it doesn't stop you from training → answer **Q8b only**.
- If you have pain that stops you from being active or playing sport → answer **Q8c only**.

8a. If you have no pain while playing sport, for how long do you train?

0 – 20 mins	20 – 40 mins	40 – 60 mins	60 – 90 mins	> 90 mins
☐	☐	☐	☐	☐
6	12	18	24	30

8b. If you have some pain while playing sport, but it does not stop you from completing your training, for how long can you train?

0 – 15 mins	15 – 30 mins	30 – 45 mins	45 – 60 mins	> 60 mins
☐	☐	☐	☐	☐
0	5	10	15	20

8c. If you have pain that stops you from playing sport, for how long can you train?

Nil	0 – 10 mins	10 – 20 mins	20 – 30 mins	> 30 mins
☐	☐	☐	☐	☐
0	2	5	7	10

TOTAL VISA SCORE ☐
