# Supplementary material for: Validity and reliability of the Dutch translation of the VISA-P questionnaire for patellar tendinopathy
Source: BMC Musculoskelet Disord. 2009 Aug 11;10:102. doi: 10.1186/1471-2474-10-102 (PMC2734752; doi:10.1186/1471-2474-10-102)
Supplement: Additional file 2 — The translated Dutch VISA-P questionnaire. The Dutch version of the VISA-P questionnaire after translation. [file 1471-2474-10-102-S2.pdf]

De Nederlandse VISA-P score

Naam: **Geboortedatum:**

Datum:

1. **Hoeveel minuten kunt u zitten zonder pijn?**

0 – 15 minuten	15 – 30 minuten	30 – 60 minuten	60 – 90 minuten	90 – 120 minuten	> 120 minuten
0	2	4	6	8	10

2. **Hebt u pijn als u de trap afloopt in een normaal tempo?**

Hevige pijn of niet mogelijk												Geen pijn
	0	1	2	3	4	5	6	7	8	9	10	

3. **Hebt u pijn als u uw knie strekt zonder er gewicht op te zetten?**

Hevige pijn of niet mogelijk												Geen pijn
	0	1	2	3	4	5	6	7	8	9	10	

4. **Hebt u pijn wanneer u een uitvalspas (lunge) maakt?**

Hevige pijn of niet mogelijk												Geen pijn
	0	1	2	3	4	5	6	7	8	9	10	

5. **Hebt u problemen met het uitvoeren van de squatbeweging (hurkbeweging)?**

Hevige pijn of niet mogelijk												Geen pijn
	0	1	2	3	4	5	6	7	8	9	10	

6. **Hebt u pijn gedurende of direct na het doen van 10 sprongetjes/hupjes op één been?**

Hevige pijn of niet mogelijk												Geen pijn
	0	1	2	3	4	5	6	7	8	9	10	

J. Zwerver, T. Kramer, I. van den Akker-Scheek 2009

7. Doet u op dit moment aan sport of andere fysieke activiteiten?

Score	☐	
0	☐	Helemaal niet
4	☐	Aangepaste training en/of aangepaste competitie
7	☐	Volledige training en/of competitie, maar niet op hetzelfde niveau als toen de symptomen begonnen
10	☐	Op hetzelfde wedstrijdniveau of op een hoger wedstrijdniveau in vergelijking met het begin van de symptomen

8. Vult u alstublieft of A, of B of C in bij deze vraag

- Wanneer u **geen pijn hebt tijdens het sporten** beantwoordt dan **alleen vraag 8A**.
- Als **u pijn hebt tijdens het sporten**, maar u hoeft **niet te stoppen** met de sportieve activiteit, beantwoordt dan **alleen vraag 8B**.
- Wanneer u **zodanige pijn hebt dat u moet stoppen met de sportieve activiteit**, beantwoordt dan **alleen vraag 8C**.

8a. Wanneer u geen pijn hebt tijdens het sporten, hoe lang kunt u dan trainen?

0 – 20 minuten	20 – 40 minuten	40 – 60 minuten	60 – 90 minuten	> 90 minuten
☐	☐	☐	☐	☐
6	12	18	24	30

8b. Wanneer u pijn hebt tijdens het sporten, maar u hoeft hierdoor niet te stoppen met sporten, hoe lang kunt u dan trainen?

0 – 15 minuten	15 – 30 minuten	30 – 45 minuten	45 – 60 minuten	> 60 minuten
☐	☐	☐	☐	☐
0	5	10	15	20

8c. Wanneer u een zodanige pijn hebt dat u moet stoppen met trainen, hoe lang kunt u dan trainen?

Niet	0 – 10 minuten	10 – 20 minuten	20 – 30 minuten	> 30 minuten
☐	☐	☐	☐	☐
0	2	5	7	10

TOTALE SCORE ☐
